# Sleep in Populations of *Drosophila Melanogaster*^1^,^2^,^3^

**DOI:** 10.1523/ENEURO.0071-15.2015

**Published:** 2015-08-21

**Authors:** Chang Liu, Paula R. Haynes, Nathan C. Donelson, Shani Aharon, Leslie C. Griffith

**Affiliations:** Department of Biology, National Center for Behavioral Genomics and Volen Center for Complex Systems, Brandeis University, Waltham, Massachusetts 02454-9110

**Keywords:** *Drosophila*, population, sleep

## Abstract

The fruit fly *Drosophila melanogaster* is a diurnal insect active during the day with consolidated sleep at night. Social interactions between pairs of flies have been shown to affect locomotor activity patterns, but effects on locomotion and sleep patterns have not been assessed for larger populations. Here, we use a commercially available locomotor activity monitor (LAM25H) system to record and analyze sleep behavior. Surprisingly, we find that same-sex populations of flies synchronize their sleep/wake activity, resulting in a population sleep pattern, which is similar but not identical to that of isolated individuals. Like individual flies, groups of flies show circadian and homeostatic regulation of sleep, as well as sexual dimorphism in sleep pattern and sensitivity to starvation and a known sleep-disrupting mutation (*amnesiac*). Populations of flies, however, exhibit distinct sleep characteristics from individuals. Differences in sleep appear to be due to olfaction-dependent social interactions and change with population size and sex ratio. These data support the idea that it is possible to investigate neural mechanisms underlying the effects of population behaviors on sleep by directly looking at a large number of animals in laboratory conditions.

## Significance Statement

Most species live in an interactive environment in their natural habitats, and sleep can be affected by social cues. Although flies have been widely used to understand the mechanisms of sleep in recent years, sleep in large populations has not been systematically studied. Here we report both similarities and differences between sleep in populations of flies compared with individuals, as well as provide a new method for the study of social behavior under constant environmental conditions.

## Introduction

Sleep has been observed throughout the animal kingdom, and performs important physiological functions that are not yet completely understood. *Drosophila melanogaster* exhibits sleep as defined by consolidated circadian periods of immobility that are associated with an increased arousal threshold. Importantly, the amount of quiescence in flies is also subject to a homeostatic regulatory mechanism (Hendricks et al., 2000; Shaw et al., 2000), suggesting that flies have a genuine sleep state. Flies have therefore been increasingly used for the neurogenetic dissection of sleep/wakefulness behavior and the circuits that produce it.

In *Drosophila*, sleep is defined as quiescence for longer than 5 min (Hendricks et al., 2000; Shaw et al., 2000). This criterion was established by examination of the timing of changes in arousal threshold after the onset of quiescence and allows measurement of locomotor activity with the standard *Drosophila* activity monitor (DAM2) system to be used to assess the amount and structure of sleep. Most previous studies of social behaviors focused on sleep by recording a pair of flies’ locomotor activity (Fujii et al., 2007; Lone and Sharma, 2012; Hanafusa et al., 2013); however, as social animals (Hay, 1973; Schneider et al., 2012), whether flies in a group sleep in a similar manner as isolated individual flies remains largely unknown. In the present study, we observed activity in larger groups in order to characterize sleep in populations of flies and compare it to individual fly sleep. We show that the commercially available *Drosophila* population monitor (LAM25H) system can be used to analyze population sleep/activity patterns and investigate the neural mechanisms of population behavior.

## Materials and Methods

### Animals

Flies were raised in a 12 h light/dark cycle on modified Brent and Oster cornmeal-dextrose-yeast agar food (Brent and Oster, 1974). Per batch: 60 L H_2_O, 600 g Agar, 1950 g flaked yeast, 1,451 g cornmeal, 6300 g dextrose, 480 g NaKT, 60 g CaCl_2_, and 169 g Lexgard dissolved in ethanol. *Canton S*, *amn^1^*, *w;Orco^2^* (also known as *Or83b^2^*; Larsson et al., 2004) and *w^CS^* flies were raised at 25°C in an incubator after eclosion. Males and mated females were used for all experiments.

### Behavioral analysis

Newly enclosed flies were raised in standard bottles and transferred to new food bottles every 2–3 d. Mating was allowed to happen freely before sorting into storage vials. Flies were sorted into small vials (50 flies per vial) 1 d prior to the loading day. Flies were 2- to 7-d-old at the start of each experiment. Individuals were placed into 65 × 5 mm glass tubes and populations of 50 flies into 95 × 25 mm glass-like vials. All sleep tubes/vials contained 2% agarose with 5% sucrose food. Flies were entrained in 12 h light/dark (LD) conditions for 2–3 d. Activity was then recorded for 2 d in LD then switched to constant darkness for another 2 d (data not shown).

For sleep deprivation (SD) experiments, a mechanical stimulus was applied using a Trikinetics plate attached to a VWR vortex mixer with a shaking frequency of 2 s of every 10 s for the entire 12 h dark period. Populations of flies housed in vials were removed from the LAM25H system onto the shaker 10 min before ZT12, and placed back immediately in the LAM25H after the 12 h of sleep deprivation to record their sleep rebound. Because data could not be collected during this period, no data are shown for the period of deprivation for the SD group. For starvation experiments, flies were transferred to 2% agarose vials at ZT0 for 24 h, and put back onto sucrose-agarose food for recovery.

### Calculation of relative sleep changes and statistical analyses

**Figure 1.**
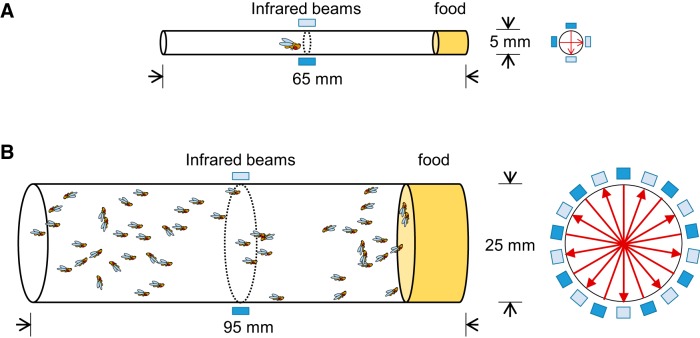
**Diagrams of DAM2 and LAM25H systems. *A***, DAM2 apparatus. Left, Side view of DAM2 sleep tube (5 × 65 mm) for individual fly recording showing location of infrared beams and food. Right, Cross-section of the tube with the orientation of the two infrared beams. ***B***, LAM25H apparatus. Left, Side view of LAM25H vial (25 × 95 mm) for population recording showing location of infrared beams and food. Right, Cross-section of the vial with the orientation of the nine infrared beams. Dark blue bars and light blue bars indicate transmitters and receivers. Red arrow lines indicate how pairs of infrared beam sensors work, as well as the coverage of the cross-sectional area.

The behavioral patterns of individuals and groups of flies were monitored using the DAM2 and LAM25H systems (Trikinetics), respectively. Diagrams of the apparati are shown in Figure 1. Sleep parameters were analyzed using an in-house MATLAB program described preciously (Donelson et al., 2012) from averages of 2 d of LD data in most experiments. All sleep manipulations (sleep deprivation and starvation) were performed for 1 d. Total sleep, number of sleep episodes, mean episode length, activity while awake, and sleep latency were analyzed for 24 h and/or 12 h light and dark periods (LP and DP). Sleep data were analyzed using Prism 6 software (GraphPad). For experiments that had multiple variables, a two-way ANOVA was performed (Table 1). Multiple comparisons after two-way ANOVA were used for each analysis period (24 h, LP and DP), and were performed to determine which pairs were significantly different and if major effects are significantly different. Holm–Sidak’s/Dunn’s test were used according to the distribution of datasets (Table 2). Datasets are marked with letters (A, B, C, or D) for statistical equivalence groups; i.e., data that are significantly different are indicated by different letters. To evaluate the sleep changes (ΔSleep) during and/or after manipulations, we subtracted the sleep during manipulation days and the sleep after manipulations from its baseline day sleep. The sleep change of the experimental group was compared with the control groups using an unpaired *t* test if it passed a normality test or Mann–Whitney test if it did not pass a normality test (Table 3). For experiments with different ratio of males in the population, datasets that did not have a normal distribution, nonparametric statistics (Kruskal–Wallis test followed by Dunn’s multiple-pairwise-comparison test) were applied. Otherwise, a one-way ANOVA followed by Holm–Sidak’s test was applied (Tables 4, 5). Figures are all presented as mean ± SEM in a uniform figure style for clarity. For single comparisons, asterisk (*) indicates a significant difference between the experimental group and the control group. The significance level of statistical tests was set to 0.05.

**Table 1.** Two-way ANOVA

						Source of variation					
						Group (inhividual vs population)		Gender (female vs male)		Interaction	
Data					DFn, DFd	*F*	*p*	*F*	*p*	*F*	*p*
Fig. 2	C		Total sleep	LP	1,76	123.7	<0.0001	86.91	<0.0001	0.02634	0.8715
				DP	1,76	31.3	<0.0001	4.876	0.0302	47.18	<0.0001
	D		Activity	LP	1,76	4077	<0.0001	138.6	<0.0001	96.81	<0.0001
				DP	1,76	162.6	<0.0001	37.59	<0.0001	18.87	<0.0001
	E		Episodes	LP	1,76	54.01	<0.0001	0.5425	0.4637	7.293	0.0085
				DP	1,76	136.5	<0.0001	6.303	0.0142	0.0005116	0.982
	F		Episode Length	LP	1,76	24.88	<0.0001	7.44	0.0079	5.683	0.0196
				DP	1,76	14.44	0.0003	0.3828	0.5379	0.5838	0.4472
	G		Latency	LP	1,76	34.82	<0.0001	24.25	<0.0001	0.008889	0.9251
				DP	1,76	20.44	<0.0001	24.45	<0.0001	3.326	0.0721

						Group (individual vs population)		Genotype (CS vs *amn*)		Interaction	
					DFn, DFd	F	P value	F	P value	F	P value
Fig. 5		C	Total sleep	LP	1,75	137.1	<0.0001	40.04	<0.0001	3.423	0.0682
				DP	1,75	15.68	0.0002	23.11	<0.0001	6.523	0.0127
		D	Activity	LP	1,75	1109	<0.0001	381.8	<0.0001	349	<0.0001
				DP	1,75	47.74	<0.0001	13.6	0.0004	15.01	0.0002
		E	Episodes	LP	1,75	15.46	0.0002	2.376	0.1274	41.79	<0.0001
				DP	1,75	329.4	<0.0001	38.07	<0.0001	0.01136	0.9154
		F	Episode Length	LP	1,75	21.08	<0.0001	7.887	0.0063	7.276	0.0086
				DP	1,75	13.87	0.0004	5.469	0.022	4.202	0.0439
		G	Latency	LP	1,75	24.08	<0.0001	13.03	0.0006	2.635	0.1088
				DP	1,75	8.588	0.0045	0.3053	0.5822	2/133	0.1483

						Gender (female vs male)		Food (complete vs sucrose agar)		Interaction	
Fig. 6		C	Total sleep	LP	1, 28	2.333	0.1379	127.2	<0.0001	33.64	<0.0001
				DP	1, 28	70.01	<0.0001	53.7	<0.0001	0.07586	0.785
		D	Activity	LP	1, 28	120.1	<0.0001	52.35	<0.0001	49.79	<0.0001
				DP	1, 28	3.479	0.0727	11.21	0.0023	4.778	0.0373
		E	Episodes	LP	1, 28	1.56	0.222	2.644	0.1151	0.7603	0.3907
				DP	1, 28	15.58	0.0005	60.54	<0.0001	41.7	<0.0001
		F	Episode Length	LP	1, 28	5.165	0.0309	23.54	<0.0001	0.8725	0.3583
				DP	1, 28	50.2	<0.0001	55.13	<0.0001	42.32	<0.0001
		G	Latency	LP	1, 28	1.581	0.219	42.69	<0.001	8.79	0.0061
				DP	1, 28	15.49	0.0005	34.96	<0.0001	26.79	<0.0001

						Gender (female vs mixed)		Size (10 vs 50 vs 100)		Interaction	
Fig. 7		C	Total sleep	24h	1,26/2,26/2,26	123.8	<0.0001	150.2	<0.0001	8.481	0.0015
		D	Activity	24h	1,26/2,26/2,26	82.69	<0.0001	86.37	<0.0001	15.01	<0.0001
		E	Episodes	24h	1,26/2,26/2,26	115.4	<0.0001	11.75	0.0002	116.8	<0.0001
		F	Latency	24h	1,26/2,26/2,26	3.249	0.0550	3.807	0.0619	2.859	0.0754

						Gender (female vs male)		Genotype (*w* vs *Orco2*)		Interaction	
Fig. 9		C	Total sleep	LP	1,21	252.2	<0.0001	56.03	<0.0001	0.1074	0.7436
				DP	1,21	34.61	<0.0001	0.4748	0.4921	11.13	0.0011

						gender (female vs male. vs Mixed)		genotype (*w* vs *Orco2*)		interaction	
		G	Total sleep	LP	2,26/1,26/2,26	281.4	<0.0001	4.992	0.0343	4.884	0.0158
				DP	2,26/1,26/2,26	117.2	<0.0001	218.9	<0.0001	80.17	<0.0001

**Table 2.** Multi-comparisons after Two-way ANOVA

	Total Sleep		Activity		Episode		Mean Episode Length		Latency	
	LP	DP	LP	DP	LP	DP	LP	DP	LP	DP
Fig. 2	Parametric	Parametric	Parametric	Parametric	Nonparametric	Nonparametric	Parametric	Nonparametric	Nonparametric	Noparametric
Female individual vs male individual	<0.0001	<0.0001	0.0339	0.0494	0.0028	0.2822	<0.0001	0.1557	0.0002	<0.0001
Female individual vs female population	<0.0001	0.3704	<0.0001	<0.0001	0.1393	0.001	0.1941	0.0155	0.3077	0.0576
Female individual vs male population	0.2067	<0.0001	<0.0001	<0.0001	0.0269	0.0073	0.2151	0.0016	>0.9999	<0.0001
Male individual vs female population	<0.0001	0.0379	<0.0001	<0.0001	<0.0001	<0.0001	<0.0001	<0.0001	<0.0001	>0.9999
Male individual vs male population	<0.0001	<0.0001	<0.0001	<0.0001	<0.0001	<0.0001	<0.0001	<0.0001	0.0016	0.1597
Female population vs male population	<0.0001	<0.0001	<0.0001	<0.0001	>0.9999	>0.9999	0.8482	>0.9999	>0.9999	0.1533
Fig. 5	Parametric	Nonparametric	Parametric	Parametric	Parametric	Parametric	Nonparametric	Nonparametric	Nonparametric	Nonparametric
CS population vs *amn1* population	0.0143	0.0246	<0.0001	0.0003	0.0001	0.0008	>0.9999	>0.9999	0.4977	>0.9999
CS population vs CS individual	<0.0001	0.9612	<0.0001	<0.0001	<0.0001	<0.0001	<0.0001	<0.0001	0.0588	0.0105
CS population vs *amn1* individual	0.0006	>0.9999	<0.0001	<0.0001	0.0007	<0.0001	0.0999	0.1171	>0.9999	0.0791
*amn1* population vs CS individual	<0.0001	<0.0001	<0.0001	0.0754	0.1481	<0.0001	<0.0001	<0.0001	<0.0001	0.3603
*amn1* population vs *amn1* individual	<0.0001	0.0107	<0.0001	0.0754	0.1481	<0.0001	0.0402	0.0043	0.0123	>0.9999
CS individual vs *amn1* individual	<0.0001	0.0158	0.3428	0.8361	<0.0001	<0.0001	<0.0001	0.0001	0.046	>0.9999
Fig. 6	Parametric	Nonparametric	Parametric	Parametric		Parametric	Parametric	Nonparametric	Nonparametric	Nonparametric
Complete female vs complete male	<0.0001	0.0363	<0.0001	0.8222	N/A	<0.0001	0.352	0.0394	>0.9999	>0.9999
Complete female vs sugar-agar female	<0.0001	0.0791	0.9001	0.0032	N/A	<0.0001	0.0016	0.0109	<0.0001	0.0002
Complete female vs sugar-agar male	<0.0001	<0.0001	0.0299	0.6616	N/A	<0.0001	0.1516	<0.0001	0.0012	0.7302
Complete male vs sugar-agar female	<0.0001	>0.9999	<0.0001	0.0048	N/A	0.0336	0.0002	>0.9999	0.0148	0.0056
Complete male vs sugar-agar male	0.0012	0.1409	< 0.0001	0.6616	N/A	0.3574	0.0388	0.559	0.0911	> 0.9999
Sugar-agar female vs sugar-agar male	0.0053	0.0681	0.0299	0.031	N/A	0.1661	0.0909	>0.9999	>0.9999	0.0535
Fig. 7	Parametric (24 h)		Parametric (24 h)		Parametric (24 h)					DP
50 female vs 10 female	<0.0001		0.0759		<0.0001					N/A
50 female vs 100 female	0.0035		0.0197		0.9473					N/A
50 female vs male+female 50	<0.0001		<0.0001		<0.0001					N/A
50 female vs male+female 10	0.0247		0.3499		0.0056					N/A
50 female vs male+female 100	<0.0001		<0.0001		<0.0001					N/A
10 female vs 100 female	<0.0001		<0.0001		<0.0001					N/A
10 female vs male+female 50	<0.0001		<0.0001		0.9473					N/A
10 female vs male+female 10	0.0066		0.3499		<0.0001					N/A
10 female vs male+female 100	<0.0001		<0.0001		<0.0001					N/A
100 female vs male+female 50	0.0007		0.0157		<0.0001					N/A
100 female vs male+female 10	<0.0001		0.0007		0.0056					N/A
100 female vs male+female 100	0.0001		<0.0001		<0.0001					N/A
male+female 50 vs male+female 10	<0.0001		<0.0001		0.0001					N/A
male+female 50 vs male+female 100	0.0123		0.0005		<0.0001					N/A
male+female 10 vs male+female 100	<0.0001		<0.0001		<0.0001					N/A
Fig. 9C	Parametric	Nonparametric								
*w* female vs *w* male	<0.0001	0.3464								
*w* female vs *Orco* female	<0.0001	>0.9999								
*w* female vs *Orco* male	<0.0001	<0.0001								
*w* male vs *Orco* female	0.0001	0.0219								
*w* male vs *Orco* male	<0.0001	0.0173								
*Orco* female vs *Orco* male	<0.0001	<0.0001								
Fig. 9G	Parametric	Parametric								
*w* female vs *w* male	< 0.0001	0.3837								
*w* female vs *w* male+female	0.8275	<0.0001								
*w* female vs *Orco* female	0.5287	0.0043								
*w* female vs *Orco* male	<0.0001	0.0012								
*w* female vs *Orco* male+female	0.1766	0.061								
*w* male vs *w* male+female	<0.0001	<0.0001								
*w* male vs *Orco* female	<0.0001	0.1477								
*w* male vs *Orco* male	0.0545	0.061								
*w* male vs *Orco* male+female	<0.0001	0.5044								
*w* male+female vs *Orco* female	0.5287	<0.0001								
*w* male+female vs *Orco* male	<0.0001	<0.0001								
*w* male+female vs *Orco* male+female	0.1105	<0.0001								
*Orco* female vs *Orco* male	<0.0001	0.6037								
*Orco* female vs *Orco* male+female	0.0156	0.5044								
*Orco* male vs *Orco* male+female	<0.0001	0.3319								

**Table 3.** t Test and nonparametric

	Data	Test	df	*t*/U	*p*
Fig. 3C	Non-SD individual vs SD individual LP	Unpaired *t* test	89	6.903	<0.0001
	Non-SD population vs SD population LP	Mann–Whitney test	30	3	<0.0001
	Non-SD individual vs SD individual DP	Mann–Whitney test	89	791	0.0554
	Non-SD population vs SD population DP	Mann–Whitney test	30	41.5	0.0007
Fig. 4C	Female nonstarved vs starved LP on starvation day	Unpaired *t* test	14	0.7764	0.4504
	Male nonstarved vs starved LP on starvation day	Unpaired *t* test	14	6.179	<0.0001
	Female nonstarved vs starved DP on starvation day	Unpaired *t* test	14	8.153	<0.0001
	Male nonstarved vs starved DP on starvation day	Unpaired *t* test	14	6.526	<0.0001
	Female nonstarved vs starved LP on recovery day	Unpaired *t* test	14	8.27	<0.0001
	Male nonstarved vs starved LP on recovery day	Unpaired *t* test	14	4.52	<0.0001
	Female nonstarved vs starved DP on recovery day	Unpaired *t* test	14	0.3369	0.7412
	Male nonstarved vs starved DP on recovery day	Unpaired *t* test	14	0.0061	0.9952

**Table 4.** One-way ANOVA and nonparametric test

	Data	Test	DFn, DFd	*F*	*p*	
Fig. 8B	24 h	One-way ANOVA	4, 27	42.35	<0.0001	
			No. of groups	No. of total values		
Fig. 8B	LP	Kruskal–Wallis test	5	32	0.0002	approximate *p* value
	DP	Kruskal–Wallis test	5	32	0.0001	approximate *p* value
Fig. 8D	24 h	Kruskal–Wallis test	6	64	<0.0001	approximate *p* value
	LP	Kruskal–Wallis test	6	64	<0.0001	approximate *p* value
	DP	Kruskal–Wallis test	6	64	<0.0001	approximate *p* value

**Table 5.** Multiple-comparisons following one-way ANOVA and nonparametric test

				24 h		LP		DP	
	Data	*n*1	*n*2	Mean (rank) differences	Adjusted *p* value	Mean rank differences	Adjusted *p* value	Mean rank differences	Adjusted *p* value
				Holm–Sidak's test		Dunn's test		Dunn's test	
Fig. 8B	Female vs male 100%	6	6	−92.42	0.3864	−6.833	>0.9999	6	>0.9999
	Female vs male 50%	6	7	388.2	<0.0001	11.31	0.3021	17.79	0.0065
	Female vs male 77%	6	7	427.3	<0.0001	12.6	0.1579	19.5	0.0019
	Female vs male 33%	6	6	435.2	<0.0001	11.83	0.2887	19.83	0.0025
	Male 100% vs male 50%	6	7	480.6	<0.0001	18.14	0.0051	11.79	0.2393
	Male 100% vs male 77%	6	7	519.7	<0.0001	19.43	0.002	13.5	0.0969
	Male100% vs male 33%	6	6	527.6	<0.0001	18.67	0.0057	13.83	0.1064
	Male 50% vs male 77%	7	7	39.07	0.7816	1.286	>0.9999	1.714	>0.9999
	Male 50% vs male 33%	7	6	46.98	0.7816	0.5238	>0.9999	2.048	>0.9999
	Male 77% vs male 33%	7	6	7.905	0.8861	−0.7619	>0.9999	0.3333	>0.9999
				Dunn's test		Dunn's test		Dunn's test	
Fig. 8D	Female vs 100% male	8	8	−24	0.1486	−44.5	<0.0001	23	0.2018
	Female vs 4% male	8	12	5.667	>0.9999	-5.5	>0.9999	8.167	>0.9999
	Female vs 10% male	8	12	22	0.1441	1.167	>0.9999	39.17	<0.0001
	Female vs 90% male	8	12	3.333	>0.9999	−22.17	0.1361	33.5	0.0012
	Female vs 96% male	8	12	-9.667	>0.9999	-31.83	0.0027	23.83	0.0754
	100% male vs 4% male	8	12	29.67	0.0072	39	<0.0001	−14.83	>0.9999
	100% male vs 10% male	8	12	46	<0.0001	45.67	<0.0001	16.17	0.8556
	100% male vs 90% male	8	12	27.33	0.0194	22.33	0.1285	10.5	>0.9999
	100% male vs 96% male	8	12	14.33	>0.9999	12.67	>0.9999	0.8333	>0.9999
	4% male vs 10% male	12	12	16.33	0.4738	6.667	>0.9999	31	0.0007
	4% male vs 90% male	12	12	−2.333	>0.9999	−16.67	0.4241	25.33	0.0128
	4% male vs 96% male	12	12	−15.33	0.6539	−26.33	0.0079	15.67	0.5883
	10% male vs 90% male	12	12	−18.67	0.2104	−23.33	0.032	-5.667	>0.9999
	10% male vs 96% male	12	12	−31.67	0.0005	−33	0.0002	−15.33	0.6539
	90% male vs 96% male	12	12	−13	>0.9999	−9.667	>0.9999	−9.667	>0.9999

## Results

### Population sleep patterns differ from those of isolated individuals

*Drosophila* are normally social animals (Hay, 1973), and their behavior and daily activity patterns can be changed by interactions with other individuals in a population (Levine et al., 2002; Krupp et al., 2008; Schneider et al., 2012). To address whether the features of sleep in populations of flies are similar to those observed for individual flies, we compared sleep patterns of isolated individual *Canton S* wild-type flies to those from groups of *Canton S* flies using data collected with the DAM2 and LAM25H systems. DAM2 records the activity of 32 individual animals using two infrared beams across a 5 mm tube (Fig. 1*A*). LAM25H records 32 vials (25 mm diameter) with high resolution using nine infrared beams across the center of each vial to detect activity (Fig. 1*B*).

**Figure 2.**
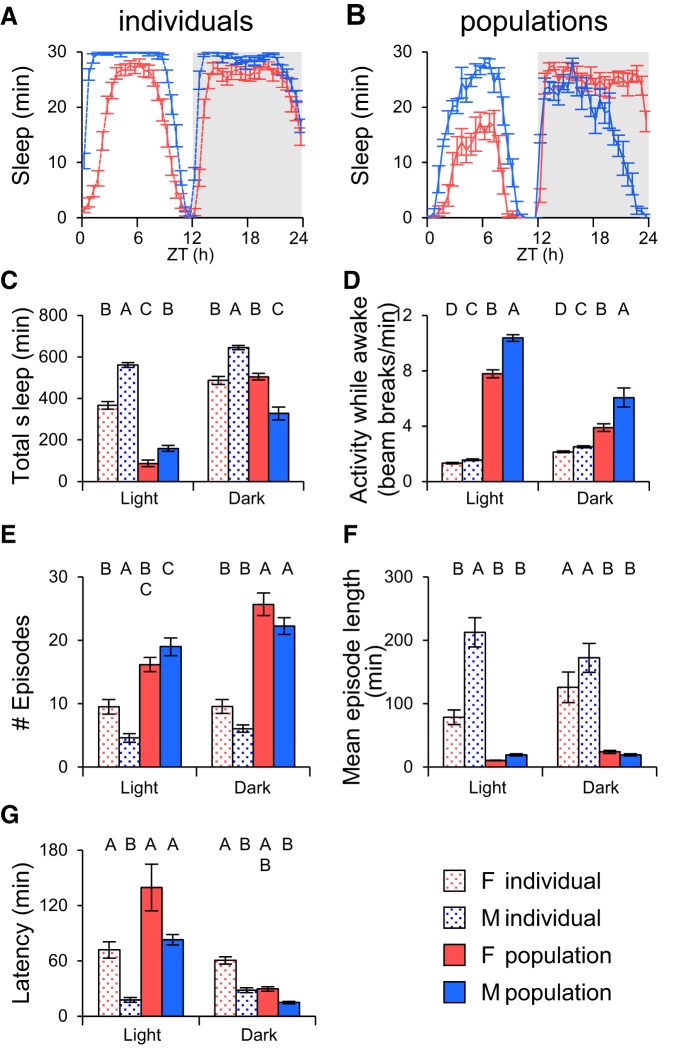
**Populations of flies exhibit sleep patterns distinct from individual flies. *A***, Individual fly sleep for males and females. ***B***, Sleep in populations of males and females. ***C***, Quantification of total sleep from ***A*** and ***B***. Individual males slept more than females. In populations males slept longer during the day, but less at night. ***D***, Activity levels during wake periods. Males had more beam breaks than females in populations. ***E***, Number of sleep episodes. Individual females had more sleep episodes than males, but populations were indistinguishable. ***F***, Mean episode length. Females had shorter episodes than individual males, but no significant difference was detected in populations. ***G***, Sleep Latency. Individual male flies took shorter time to fall asleep after light transitions than individual female flies, but no significant difference was found between populations of males and females. *n* = 32 for individuals and *n* = 8 groups for populations. Statistically similar groups are marked by the same letter, with different letters indicating significant differences between groups. F, female; M, male; ZT, Zeitgeber time.

As shown in Figure 2*A* and *C*, individual males slept more than individual females during the day, consistent with previous reports of sexual dimorphism (Huber et al., 2004; Andretic and Shaw, 2005; Isaac et al., 2010). When kept in same-sex groups, daytime sleep was still greater in males than in females, but the total amount of sleep for both sexes was lower (Fig. 2*B*,*C*) suggesting that perhaps the presence of other flies and the attendant sensory stimulation may decrease sleep.

A more interesting difference between individuals and groups was seen in nighttime sleep. When flies were alone, males slept more than females at night (Fig. 2*A*). In same-sex populations, however, males slept significantly less than females and this difference was mostly due to a decrease in sleep in the last half of the night (Fig. 2*B*). The total amount of nighttime sleep compared between individuals and same-sex groups was the same for females, but significantly less for males (Fig. 2*C*). These data imply that male–male interactions either increase late at night or are more arousing in that time window than female–female interactions.

The differences in sleep were not due to sex-dependent differences in locomotor activity. Activity levels during waking periods were higher in males than in females for both isolated individuals and groups of flies, and activity counts during waking periods were higher for groups than for isolated flies for both sexes (Fig. 2*D*). Because an activity count (beam break) can be generated by a single fly, groups will naturally have more beam breaks during active periods and this will lead to an increase in overall “activity”. Activity during wake periods in the population datasets is therefore not a measure of locomotor activity or speed of individuals in the population, it is an aggregate measure that reflects both individual locomotor activity level and the number of flies that are active in a time window. If the presence of other flies stimulates locomotion differentially at different times of day, this would manifest as differences in activity but would not necessarily mean that individual flies were moving faster/slower at that time of day. The difference between male and female groups suggests that there are sex-specific increases in population activity that might be caused by interactions between males. We speculate that this might reflect increased aggression, but without direct observation it is difficult to know. In any case, this difference in basal activity likely contributes to the differences in nighttime sleep between same-sex groups.

Sleep architecture metrics were also affected by sex and group interactions. Individual male flies exhibit more consolidated sleep than individual female flies; i.e., fewer episodes but longer episode duration. In populations of flies, however, there was no difference between male and female groups (Fig. 2*E*,*F*). The increase in number of episodes and the decrease in their duration for groups compared with individuals is likely a reflection of the fact that during a population sleep episode all flies in the group must necessarily be immobile, but the activity of a single fly in the group can terminate a population sleep bout. The calculated average population sleep bout duration therefore reflects the minimum sleep bout duration for individuals in the group rather than an actual average length, which is an important distinction in interpreting population data. In general, sleep structure parameters for individuals cannot be extrapolated from population data in a quantitative manner.

In contrast to total sleep and sleep structure parameters, latency to sleep appeared to scale similarly between isolated flies and groups. Isolated males had shorter latency to sleep onset than isolated females, but there were no significant differences between grouped males and females (Fig. 2*G*). The absolute latency to sleep onset was much higher in groups during the day, whereas nighttime latencies were of similar magnitude for both individuals and groups. This may reflect a difference in sleep drive during the day and the night. At night, sleep drive is strong enough to overcome the sensory stimulation provided by other individuals in the group, but during the day these sensory inputs are disruptive in groups.

These results suggest that quiescence in populations of flies shares similarities in overall presentation with sleep that has been characterized in individual animals in terms of day/night distribution and sexual dimorphisms in the amount of daytime sleep. The fact that same-sex groups show qualitative differences in sleep patterns and activity during the night, however, implies that the presence of other animals affects sleep in ways that are not simply due to changes in the number of flies in the apparatus. Sex-specific social interactions appear to modulate the amount and pattern of nighttime sleep.

### Homeostatic sleep regulation in populations of flies

**Figure 3.**
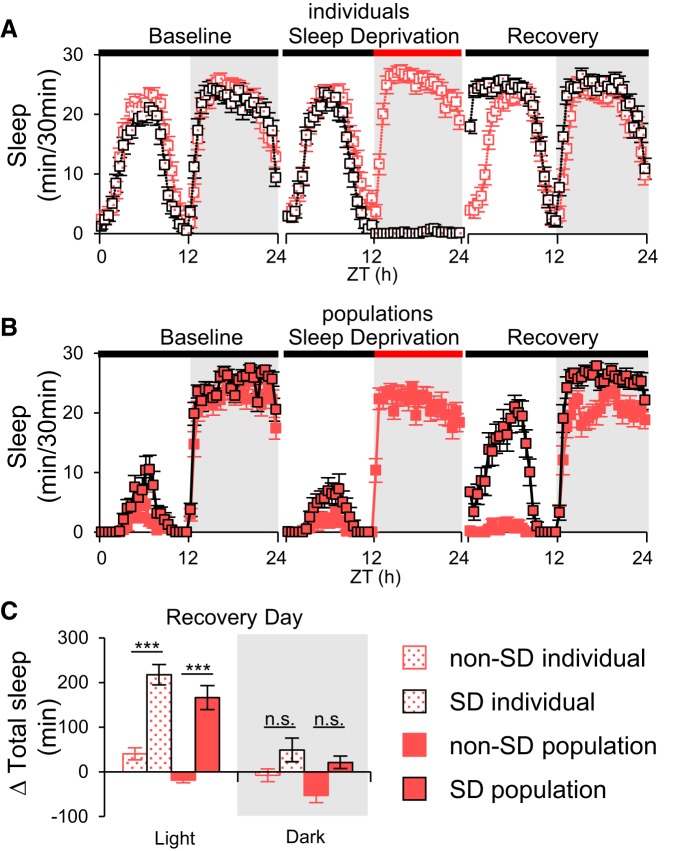
**Populations exhibit homeostatic rebound sleep after mechanical sleep deprivation. *A***, Sleep profiles of individual female flies (*n* = 48 and *n* = 43 for non-SD and SD, respectively) were recorded using DAM2. ***B***, Sleep profiles of groups of 50 female flies (*n* = 16 groups for both non-SD and SD) were captured using LAM25H. Red bar indicates the sleep deprivation period in both experiments. In ***B***, the absence of data points for the SD groups during the SD period is because of the need to remove the population vials from the monitor during shaking (see Materials and Methods). ***C***, Quantification of recovery day sleep. Day time sleep increased significantly on the recovery day after 12 h of sleep deprivation by mechanical shaking. Sleep changes were normalized to the baseline day. Δ Total sleep: total sleep changes. ZT, Zeitgeber time; SD, sleep deprivation. ***p < 0.0001; n.s., no significant difference.

**Figure 4.**
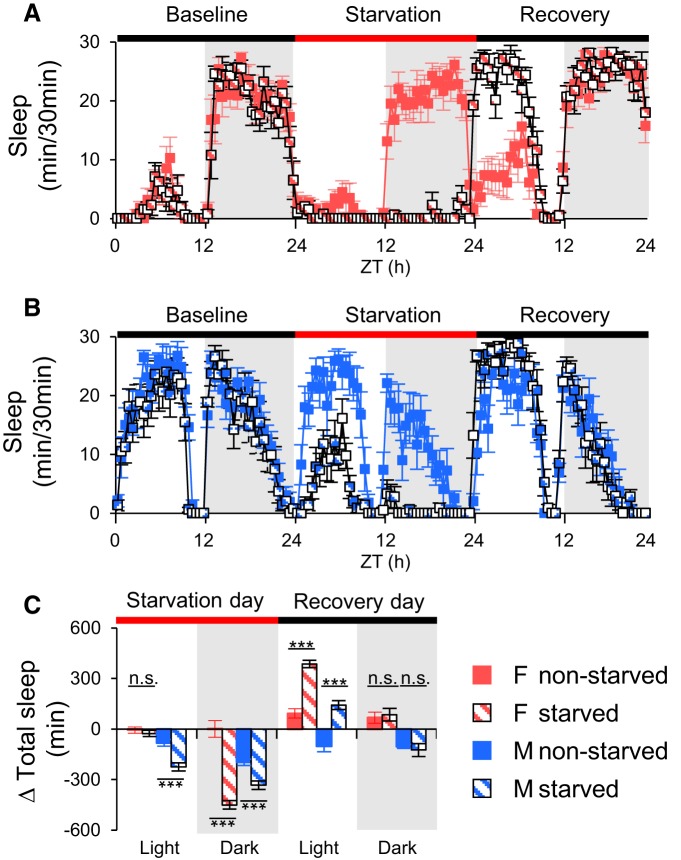
**Suppression of sleep by starvation generates rebound sleep in populations.** Sleep patterns generated by starvation in female (***A***) and male (***B***) flies in populations. ***C***, Total daytime and nighttime sleep changes are plotted as mean ± SEM. Male flies’ sleep was reduced significantly during the day and night, but female flies’ sleep was significantly suppressed only in the night. Red bar indicates the starvation period. Twenty-four hour starvation-induced sleep loss was compensated after feeding on the recovery day. Δ Total sleep: total sleep changes. *n* = 8 for all conditions. ***p < 0.0001; n.s., no significant difference. ZT, Zeitgeber time; F, female; M, male.

Although populations demonstrate quiescence periods with the same basic structure and many of the properties of sleep that have been characterized in individual flies, to be considered true sleep, this quiescence has to be homeostatically regulated. To examine this issue we used two methods for disrupting sleep. First, we mechanically deprived female flies of sleep for 12 h overnight and measured the amount of excess sleep that was produced over the following 24 h. Both isolated females and groups of females had a significant amount of homeostatic rebound sleep during the day following sleep deprivation (Fig. 3). As a second method of sleep deprivation, we used starvation (Keene et al., 2010) to deprive both males and females. We used a 3 d protocol to monitor changes in sleep during and after 24 h of food deprivation. We found that male flies’ sleep was significantly reduced in both day and night, but female flies’ sleep was significantly suppressed only at night (Fig. 4*A*–*C*). Twenty-four hour starvation-induced sleep loss was compensated after feeding on the recovery day (Fig. 4*C*). Both with mechanical and starvation-induced sleep deprivation, rebound sleep in populations occurred primarily during the light period of the recovery day, consistent with previous reports (Shaw et al., 2000). The fact that we can see enhanced sleep after two different methods of deprivation supports the notion that the inactivity we measure in populations is associated with true sleep.

### Population sleep is disrupted by mutation of *amnesiac*

It is clear that quantitative parameters, such as bout length and number in population sleep data, cannot quantitatively reflect the architecture of sleep of individuals in a group due to the manner in which the locomotor data are acquired (see above). To determine whether these measurements can qualitatively inform our understanding sleep architecture, we compared population sleep in the *amnesiac* mutant, which is known to have disrupted sleep structure (Liu et al., 2008), with *Canton S* wild-type to see if population measurements would be able to capture the previously characterized defects.

**Figure 5.**
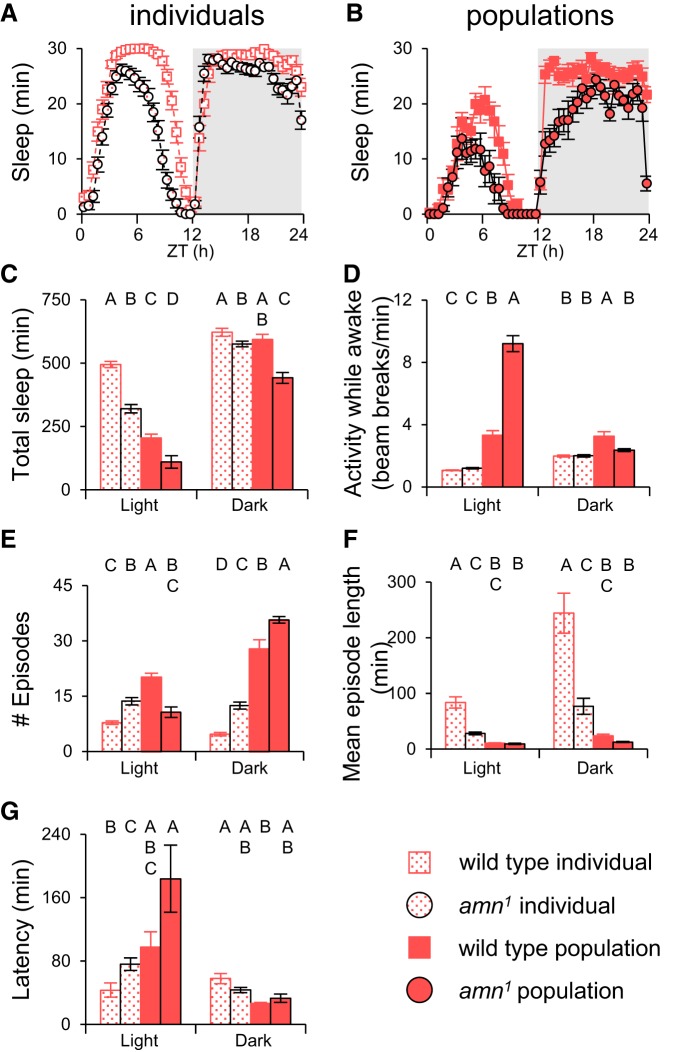
***amn^1^* Mutant flies housed in populations show a fragmented sleep pattern, similar to that of *amn^1^* individuals.** Sleep profiles of *amn^1^* mutant flies compared with wild-type *Canton S* flies in individuals (***A***) and populations (***B***), respectively. ***C***, Quantification of data. *amn^1^* mutant flies slept less than wild-type flies in population as well as individuals. ***D***, Activity during waking. Populations of mutant flies were hyperactive during the light period but hypoactive in the dark compared with controls; however, no difference was detected in individual flies. ***E***, Number of sleep episodes. Sleep episodes increased significantly in populations of *amn^1^* mutant flies compared with controls at night consistent with individuals, but exhibited the opposite phenotype during light period. ***F***, Episode length. Populations of mutant flies did not show significant difference in sleep episode length where individual mutant flies decreased dramatically compared to wild-type. ***G***, Latency. *amn*^1^ mutant flies exhibited similar latency compared to wild type flies at night in both individuals and populations. *n* = 8 groups for both wild-type and *amn^1^* populations. *n* = 31 and *n* = 32, respectively, for wild-type and *amn^1^* individuals. ZT, Zeitgeber time.

Similar to what had been reported for *amn^X8^* mutants (Liu et al., 2008), we observed loss of sleep both during the day and night, and significant sleep fragmentation in isolated *amn^1^* females. *amn^1^* Mutant female populations also showed significantly lower sleep during the day and night, and a significantly increased number of sleep episodes at night compared with the wild-type control (Fig. 5). No difference was found between populations of *amn^1^* mutants and wild-type flies in episode length, but absolute episode length of populations is very short compared with that of individuals (Fig. 5*E*), perhaps reflecting a floor effect. Surprisingly, the number of sleep episodes of populations of *amn^1^* flies during the day time actually decreased compared with wild-type, going in the opposite direction from individual fly measurements (Fig. 5*F*).

This difference in the number of episodes may be due to changes in locomotor activity. Individual *amn^1^* mutants did not differ in locomotor activity level during wake periods from wild-type flies; however, populations of *amn^1^* mutants exhibited increased activity during the daytime while they were awake and decreased activity at night during wake periods (Fig. 5*D*). As discussed above this could reflect either changes in individual fly locomotion or could reflect changes in the number of flies active during these time windows. In either case it suggests that the *amn* gene might have a specific role in the responses to social situations that is not seen in isolated animals. One possibility is that interactions with other flies cause *amn* mutants to become hyperaroused during the day. *amn* Mutants lack ability to focus selective attention on visual stimuli (Wu et al., 2000) and have an exaggerated locomotor response to ethanol (Wolf et al., 2002), consistent with altered regulation of arousal (Chi et al., 2014). Until this is experimentally addressed, however, this conclusion remains speculative.

In contrast to a previous study (Liu et al., 2008), we found that *amn^1^* mutants appeared to have no change in nighttime sleep latency compared with wild-type flies in either individual or population measurements (Fig. 5*G*). The differences between our study and that of Liu et al. (2008) may arise from a number of factors. We used an *amn^1^* stock which was outcrossed to w+ *Canton S*, whereas Liu et al. (2008) used *amn^X8^* on a *w* background. The differences in latency effects may be allele- or genetic background-specific. Our latency results with *amn^1^* are qualitatively similar to what we observed with wild-type flies (Fig. 2), which supports the idea that very high sleep drive can overcome differences in arousal state caused by genotype and the presence of other individuals.

### Food quality alters sleep patterns in populations of flies

**Figure 6.**
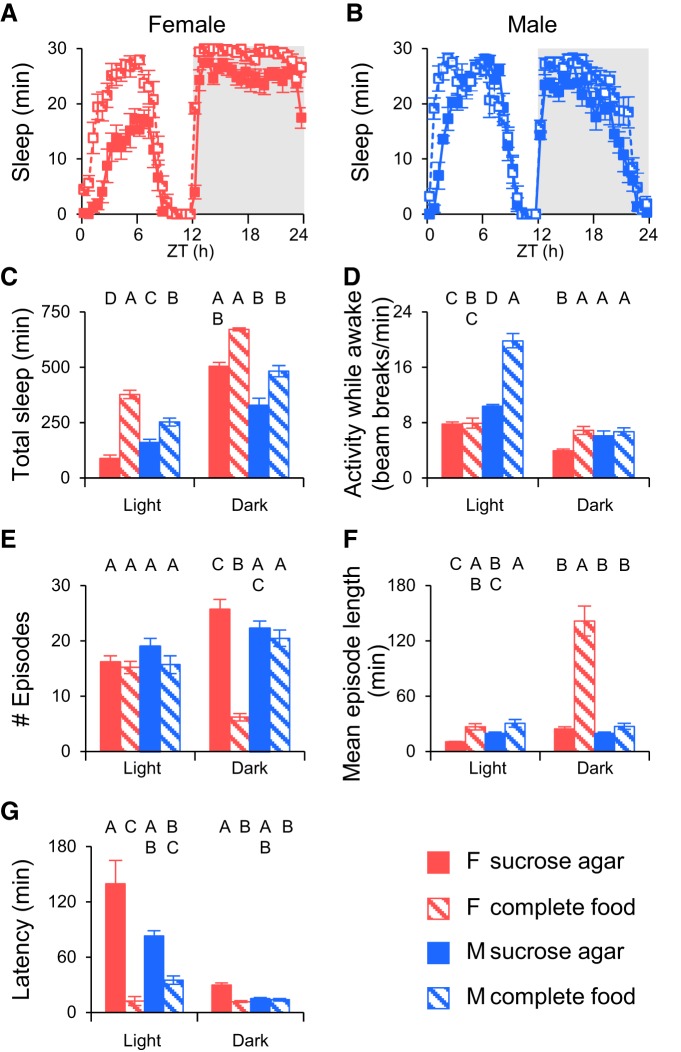
**Populations of flies sleep better with complete food.** Sleep profiles for populations of females (***A***) and males (***B***) on different food. ***C***, Quantification of total sleep. Both female and male populations of flies slept significantly longer when on standard fly food compared with sucrose during the day, but there was no statistically significant difference during the night. ***D***, Activity while awake. Complete food significantly increased activity levels during daytime wake periods in males, and at night in females. ***E***, Number of sleep episodes. ***F***, Sleep bout length. Females, but not males, had significantly consolidated sleep at night, i.e., fewer but longer sleep episodes. ***G***, Latency. Females fell asleep faster on the complete food than on the sucrose agar food, whereas males exhibited similar latency on both food media. *n* = 8 groups for all conditions. ZT, Zeitgeber time; F, female; M, male.

Although many mutants, such as *amn*, have been shown to affect sleep, environmental factors such as food quality can also have a profound influence (Zimmerman et al., 2012). To examine whether food quality influences population sleep parameters, we examined sleep patterns on either standard complete food or sucrose-agar food (see Materials and Methods for food details) and compared wild-type *Canton S* females and males. Both female and male groups slept significantly longer on standard food compared with sucrose only during the day (Fig. 6*A*–*C*). Interestingly, standard food resulted in a significantly more consolidated nighttime sleep pattern (fewer sleep episodes but longer episode duration) in groups of females but in not groups of males (Fig. 6*E*,*F*). With standard food, activity during wake periods was significantly elevated in males during the day and in females at night (Fig. 6*D*), suggesting an interaction of food quality and sex on locomotor activity. Populations of females synchronized their sleep on standard food faster than on sucrose-agar food after lights on/off, but no significant difference was detected in males (Fig. 6*G*). Altogether these results suggested that food quality has a sex-specific impact on population sleep parameters.

### Social interactions alter population sleep

**Figure 7.**
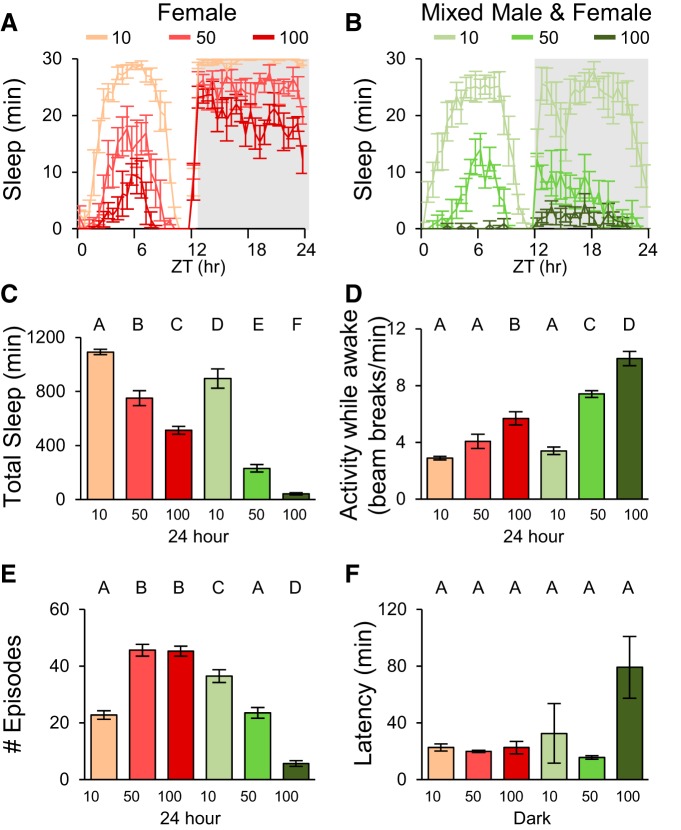
**Sleep is affected by population size and social behavior. *A***, Sleep profiles for female populations of different sizes. ***B***, Sleep profiles for populations of males and females (1:1 ratio of sexes). ***C***, Quantification of total sleep. Total sleep was decreased significantly with increasing number of flies and mixed populations with the same number of total flies exhibit lower sleep than populations of female flies. ***D***, Activity while awake. Increasing the number of flies increases population activity. ***E***, Number of sleep episodes. The number of episodes scales with population size in opposite directions for female only and mixed populations. ***F***, Sleep latency does not change significantly with population size. *n* = 5–6 groups for all conditions. ZT, Zeitgeber time.

All the data shown in previous figures used a population size of 50 individuals of the same sex. To determine whether population sleep parameters are correlated with population size and to test the effect of mixing the sexes, we measured sleep in all-female and 1:1 male–female mixed populations of 10, 50, and 100 individuals (Fig. 7). Increasing the number of flies dramatically decreased total sleep (Fig. 7*A*–*C*), and also increased locomotor activity during wake periods (Fig. 7*D*) for both female and mixed groups. This is consistent with increased population density providing a higher level of sensory input and consequent arousal. Interestingly, daily sleep episodes exhibited opposite trends in female and mixed populations. Increasing the number of females in a group increased the number of episodes, whereas in mixed populations the number of episodes decreased with increasing population size (Fig. 7*E*). Nighttime sleep latency was neither significantly affected by the number of flies nor by gender of the population (two-way ANOVA, gender: *F*_(1,26)_ = 3.294, *p* = 0.0550; size: *F*_(2,26)_ = 3.807, *p* = 0.0619; interaction: *F*_(2,26)_ = 2.859, *p* = 0.0754; Fig. 7*F*), suggesting sleep drive can overcome the arousing effects of increased population size.

**Figure 8.**
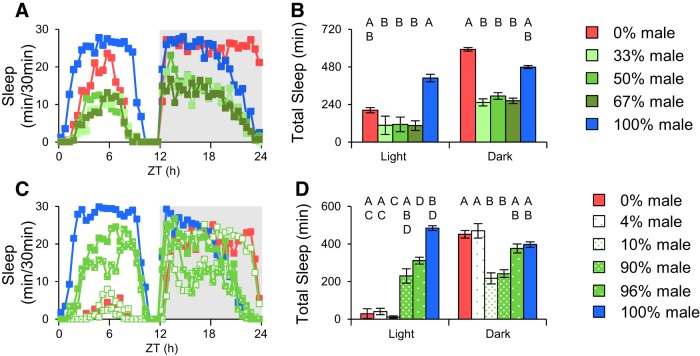
**The ratio of male–female flies in mixed populations affect total sleep.** Two experiments were done to test the effects of changes in sex ratio. ***A***, Different ratios from 0 to 100% male were tested. Data are quantified in ***B***. ***C***, Small changes in ratios around the extremes were tested. Data are quantified in ***D***. Mixed populations of flies had generally lower sleep than female or male same sex populations. Small changes in the number of males or females affected sleep most significantly at the extremes. To view sleep profiles clearly, error bars were omitted from ***A*** and ***C***. *n* = 5–7 groups for all conditions. ZT, Zeitgeber time.

A previous study looking at pairs of flies showed that the locomotor activity pattern is driven dominantly by males (Fujii et al., 2007). To further address whether the ratio of males to females in a group has an impact on population sleep, we compared female and males with a ratio of 1:1 (50% male), 1:2 (67% male), and 2:1 (33% male) to same-sex male and female populations. Total daily sleep in all three mixed groups were equivalent, about one-half that of the single-sex groups (Fig. 8*A*,*B*), indicating that total sleep was not sensitive to changes in sex ratio around equivalence. Interestingly, the pattern of nighttime sleep in all these groups looked very much like that of males, with a sharp drop in sleep during the last one-half of the night (Fig. 8*A*). To look at more skewed ratios farther from equivalence we monitored sleep in groups of 50 flies that were 4, 10, 90, and 96% male. Interestingly, 10% males in a female-dominant group could have a significant effect on sleep, driving it into a male-like pattern at the end of the night (Fig. 8*C*,*D*). These results suggested that the sexual interactions play a role in regulating population sleep.

**Figure 9.**
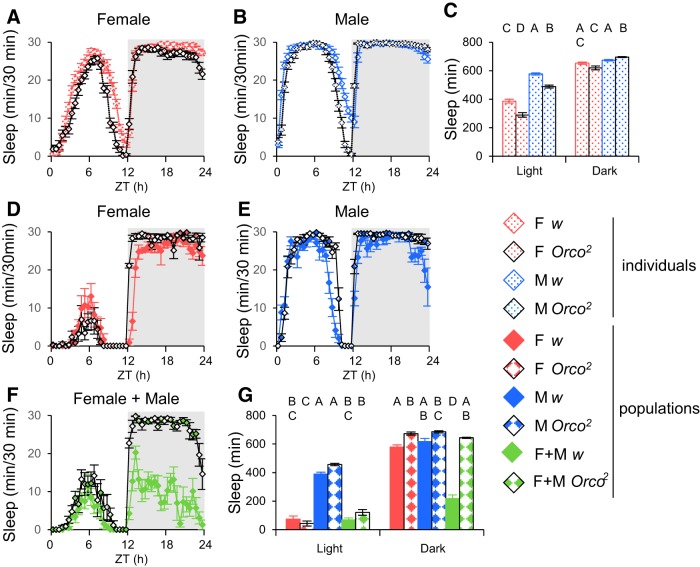
**Olfactory input modulates sleep amount by influencing social interactions.** Sleep profiles of individual female (***A***) or male (***B***) flies. ***C***, Quantification of data. *Orco^2^* mutants slept significantly less than *w* controls during the day, and male *Orco^2^* mutants slept longer than *w* males at night. No significant difference was detected between individual female *Orco^2^* mutants and *w*. *n* = 30–32 for all genotypes. Population sleep profiles for female (***D***), male (***E***), and 1:1 mixed-sex populations (***F***). ***G***, Quantification of data. Total sleep in populations of *Orco^2^* mutant flies was similar to *w* controls within the male and female groups during the day and night. However, mixed female and male populations of *Orco^2^* mutants exhibited drastically elevated sleep compared with *w* controls during the night. *n* = 5–6 groups for all genotypes. ZT, Zeitgeber time; F, female; M, male.

Previous studies on mixed-sex pairs of flies have shown that courtship, a behavior that can be driven by olfactory cues, likely plays an important role in increased nighttime locomotor activity (Fujii et al., 2007; Hanafusa et al., 2013). To determine whether the social interaction influences on sleep we have seen were mediated via olfactory input, we compared isolated animal and population sleep for the olfactory receptor mutant *Orco^2^* (also known as *Or83b^2^*) with a *w* genetic background control line in normal LD. For individual flies (Fig. 9*A*–*C*), olfaction affects sleep similarly in males and females. During the day, flies with compromised olfaction slept less than *w* control flies. At night, the main effect of genotype (*Orco^2^* mutant, *w* control) on total sleep was not significant (two-way ANOVA, *F*_(1,21)_ = 0.4748, *p* = 0.4921). Overall, these results suggest a mild sleep-promoting effect of olfaction on isolated flies.

In populations (Fig. 9*D*–*G*), *Orco^2^* mutant generally slept more at night, suggesting a role for olfactory input in the suppression of sleep in populations. The biggest effects were seen in mixed-sex populations. *Orco^2^* mutants failed to decrease nighttime sleep (Fig. 8*F*,*G*), suggesting the change in sleep in mixed-sex groups reflects olfaction-dependent, perhaps sexual or aggression-related, behavior. Interestingly, *Orco^2^* mutants also lacked the late-night male-specific decrease in sleep seen (with varying magnitude; Zimmerman et al. 2012) in *Canton S* male populations (Fig. 2*B*) and *w* control male populations (Fig. 9*E*) supporting the idea that this might reflect an olfactory-driven male–male interaction like aggression (Liu et al., 2011).

## Discussion

Mechanisms of sleep have been studied widely; however the effects of social context on the characteristics of sleep have not been systematically evaluated. In the studies we report here, we provide evidence that sleep occurs in populations of flies through assessment of sleep patterns and homeostasis. We find that sleep patterns in populations of flies are distinct from those of individual flies and that many of these differences are likely to be rooted in the effects of social interactions on sleep.

The utility of the fruit fly in the study of population sleep depends on whether a group of flies can synchronize their sleep/wake activity. In this respect we find that populations of flies behave in the same manner as individuals. They exhibit the same morning and evening activity peaks, as well as having a siesta during the light period and more sleep during the dark period. In addition, and consistent with previous observations (Hendricks et al., 2000), we found that populations of flies fall asleep quickly, within the first hour after lights off with both female and male populations having similar sleep onset (Fig. 2*G*). These phenomena are unaffected by population size or gender. Importantly, we also find that the quiescence we see in populations is under homeostatic control, an important criterion for inactivity to be called “sleep” (Huber et al., 2004; Cirelli and Bushey, 2008). Sleep deprivation generated by mechanical shaking or by starvation induced recovery sleep in populations of flies (Figs. 3, 4).

A critical question for these studies is whether the population activity measurements we present quantitatively reflect sleep in individuals within the population. Population sleep has a number of characteristics which suggest that what we are measuring is rooted in the sleep behavior of the individuals in the group; i.e., it is packaged as circadian clock-regulated periods of immobility and it is homeostatically regulated. But the quantitative relationship of population and individual measurements is more complicated. All the metrics previously used to characterize sleep are based on measurements of the behavior of individuals and we are measuring the activity of the population as a whole. What we can say about LAM25H data is that when the population is “asleep”, every individual by definition must also be because there are no beam breaks. What we cannot determine is what waking activity in the population means: is one fly active or all the flies active? Video recording of populations did not reveal any obvious cases of single flies “driving” wake activity in wild-type populations (data not shown) but this would have to be more rigorously examined for other genotypes to rule it out completely.

There is also a strong caveat to interpretation of sleep architecture data from populations of flies. Sleep fragmentation is usually assessed in individual animals by looking at the number and length of sleep bouts. In population data, a sleep episode is a period during which all the flies are inactive, but an episode can be terminated by the activity of a single fly. This means that the bout duration in population data reflects a minimum value for the sleep of individuals in that population. Together, this implies that population measurements are likely to be underestimates of the sleep of individuals in that population and that the interpretation of sleep structure measurements is necessarily different.

To really look quantitatively at the effects of being in a group on an individual’s sleep pattern, one would need a system where the activity of individuals could be monitored in the context of the population, but for the densities of flies we are looking at here and the geometry of the arena (a vial), that is likely to be quite difficult even with currently available tracking software (Branson et al., 2009; Swierczek et al., 2011; Ardekani et al., 2012). Most of these tracking systems are based on capture of two-dimensional images which distinguish subjects from the background and determine their path. This would be difficult to do in a 3D arena, such as a vial. Ardekani et al. (2012) developed a 3D movement-tracking system by using multiple cameras positioned around a vial to track freely-moving GFP-labeled flies, but application of this approach to studies such as ours would be hindered by the necessity of having GFP in all genotypes and the complexity of the instrumentation for doing large-scale experiments where data needs to be collected simultaneously from many vials.

In spite of these shortcomings, population measurements using the LAM25H system are able to recapitulate findings that have emerged from single-fly studies on mutant and environmental effects on sleep. Many genes have been identified which influence sleep (Cirelli et al., 2004; Wu et al., 2008). A previous study suggested *amn* plays a major role on sleep architecture (Liu et al., 2008). In our study, populations of *amn^1^* mutants exhibited fragmented sleep at night with significantly increased number of sleep episodes and an overall reduced amount of sleep, similar to individual flies (Fig. 5). In addition, we observed populations of *amn^1^* flies had a novel locomotor phenotype with hyperactivity during the day and hypoactivity at night. We speculate that this may reflect a role for *amn* in social interactions that has not been previously reported, but is consistent with reports of hyperarousability to other stimuli (Wolf et al., 2002).

Population sleep measurements also are able to detect the suppressing effects of starvation on sleep similar to those that have been previously reported. Starvation has been shown to induce sleep loss during both the day and night in individual females but only at night in individual male flies (Keene et al., 2010). Interestingly, in our study, male flies housed in populations had an immediate decrease in sleep (Fig. 6*B*), whereas female populations only suppressed sleep after 12 h (Fig. 6*A*), suggesting that sleep suppression is sexually dimorphic, perhaps dependent on metabolic effects of starvation, social interaction or possibly survival competition within a group. In contrast to a previous study which found no effects of starvation on sleep homeostasis in individual flies (Thimgan et al., 2010), we found a robust sleep rebound in populations after starvation-induced sleep loss, providing a potential method to investigate homeostatic regulation by feeding state. We can also capture effects of food quality on sleep in populations. Although a previous study suggested sufficient caloric intake with no amino acids was able to support normal levels of sleep in individuals (Keene et al., 2010), we observed that populations of flies housed with sucrose food had less sleep than those given complete food. Housing in populations may exaggerate an effect that was undetectable in individuals, or increased activity of populations may generate more metabolic need. In contrast to a previous study (Zimmerman et al., 2012), which found increased total sleep/consolidated sleep when flies were switched to sucrose-based food from molasses- or dextrose-based food, we observed the opposite phenotypes in female populations. This might be due to differences in genetic background, mating status, or social context. Interestingly, we observed that sucrose food did not influence sleep architecture in male populations, whereas females robustly increased the number of sleep episodes and reduced sleep episode length during the dark period. In a previous study (Linford et al., 2012), diet was shown to alter sleep architecture such that both individual male and female flies exhibited an increasing number of shorter sleep episodes with a low dietary sugar (5%) compared to higher sugar medium. Our results support the idea that the nutritional environment has an impact on sleep behavior, but suggest that these effects are sex-specific in a group context. The ability of population sleep measurements to find similar effects of mutations and nutritional state on sleep as have been reported for individuals supports the idea that they represent sleep at least at a qualitative level.

The quantitative differences between population and individual sleep are likely a function of social behaviors. Social interactions have been demonstrated to influence rhythmicity in humans (Stern and McClintock, 1998), rodents (Mrosovsky, 1988), bees (Toma et al., 2000), as well as in flies (Levine et al., 2002; Schneider et al., 2012). In *Drosophila*, it has been shown that the clocks of group-housed individuals are more synchronized than animals that have been isolated (Levine et al., 2002). In our study, we also observed that populations of flies (regardless of whether they were male, female, or mixed) synchronized sleep onset very quickly, within 1.5 h of lights off (Fig. 7*F*). The synchronized sleep/wake pattern in populations of flies may be due to the same volatile chemical signals which synchronize locomotor activity (Levine et al., 2002; Lone and Sharma, 2011), though the ability of *Orco^2^* mutant flies to show similar synchronization suggests that these volatile signals may be detected by ORCO-independent olfactory pathways or that other cues can also be used to synchronize behavior.

There are also likely to be direct courtship- and mating-related effects on sleep. Courtship activity has been shown to be higher during the night and morning (Sakai and Ishida, 2001; Tauber et al., 2003; Hanafusa et al., 2013) and male flies play a dominant role in nocturnal locomotor activity in pairs of flies (Fujii et al., 2007). Male sex peptide, which is transferred to females when they mate, can inhibit female daytime sleep (Isaac et al., 2010). Reproduction-specific roles in regulating group dynamics could be a critical biological function in ecological contexts. Our sleep data showing drastically decreased sleep in the last half of the night in mixed populations also suggest the possibility that this is a time window during which mating occurs.

Many studies have shown that olfaction is a major driver of social interactions in flies (Levine et al., 2002; Fujii et al., 2007; Lone and Sharma, 2012). In this study, we find that populations of *Orco^2^* mutant flies have no difference in total sleep compared with wild-type populations during the day, suggesting that other sensory input can compensate for the loss of olfaction for the synchronization of daytime activity. At night, however, loss of olfactory input, especially in male–female mixed populations, increases sleep time. This suggests that social interactions regulated by olfaction, such as courtship and aggression (Wang and Anderson, 2010; Liu et al., 2011; Dweck et al., 2015) specifically affect nighttime sleep. Although other social stimuli (sight, sound, mechanosensation, etc.) could also influence nighttime behavior, olfaction appears to have an important role.

In summary, we find that sleep can be measured in populations of flies, but the characteristics of population sleep vary from those of individual sleep. Some of the differences are due to technical considerations, e.g., the interpretation of sleep structure metrics from populations, but other reflect major effects of social interactions on sleep. Our demonstration of sex-specific and olfaction-related changes in sleep will provide interesting new avenues for understanding social behavior.

## Figures and Tables

**Figure:**
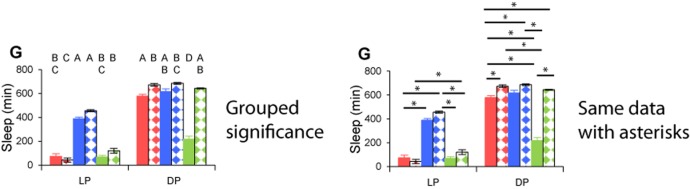


**Figure:**
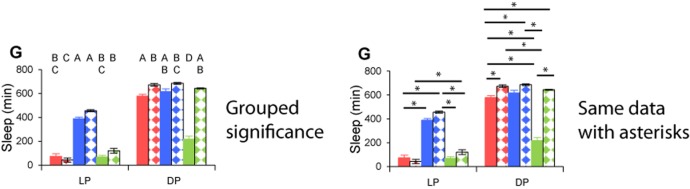


## Synthesis

The decision was a result of the Reviewing Editor Vivian Budnik and the peer reviewers coming together and discussing their recommendations until a consensus was reached. A fact-based synthesis statement explaining their decision and outlining what is needed to prepare a revision is listed below. The following reviewer(s) agreed to reveal their identity: Dennis Mathew

Synthesis Statement for Author (Required):

Both reviewers agree that the manuscript would be an important addition to the Drosophila community studying sleep. They concur that the manuscript should be accepted with minor revisions requiring editor's review. The required revisions are as follows.

1. There should be a better description of the paradigm used (including diagrams of the set up, and an account of software routines used for analysis), with explicit discussion of caveats for each conclusion reached.

2. Use asterisks consistently throughout the figures to denote significance, following eNeuro rules.

3. Discuss the discrepancy with the notion that increased total sleep is accompanied by lower activity in Fig. 5C and D.

4. Use a multiple color scheme to increase the readability of Fig. 6A, B

5. There are a few unsupported claims (e.g. link between aggression and diminished sleep; hyperarousal in amn mutants), which should be properly placed as speculative statements or eliminated.

6. There are several other useful comments by reviewers (e.g. construction of a "sleep space" plot for comparisons, rewrite last sentence of abstract, discussion of sleep time calculation, etc) that would improve the manuscript and these should be considered by authors.

Reviewers comments:

Reviewer 1:

In this study, the authors record and analyze sleep behavior in a population of fruit flies. The authors suggest that there are both similarities and differences in sleep characteristics between individual flies and a population of flies. They also suggest that the LAM25H system can be used as a method to investigate neural mechanisms of population behavior.

Admittedly, the described technique to measure sleep in fly populations has its own limitations, especially in the interpretation of sleep structure metrics from populations. If some of the underlying concerns of data interpretation are resolved, this study would be a step forward in understanding the dynamics of sleep in a population of animals. Overall, the study described in this ms. is one that would be useful to the Drosophila sleep community.

Below, I have listed some major and minor concerns with this ms. These include some questions/suggestions that the authors might consider to further improve the ms.

Major concerns;

1. I can't help wonder whether activity in a population of flies truly reflects population behavior or whether it is simply the result of activity of one or few flies that are outliers. The authors admit that their behavioral paradigm cannot distinguish between these possibilities and this is a serious limitation of the paper. However, I am convinced that the presented evidence (especially the sex-specific differences in populations of flies) strongly suggest that population effects on sleep are real. Perhaps, the ms. would greatly benefit with a better description of the paradigm and a better explanation of the caveats in the results. For instance, the authors mention that there are 9 beams across the center of each vial to detect activity, but no explanation is given as to how this achieves a better resolution of fly locomotion and population behavior. A diagram of the paradigm might be a useful addition.

2. In my opinion, the most interesting result in this study has to deal with the population effect on sleep in male flies. Perhaps the ms. would be less complex to follow and more interesting if the subsequent analyses are focused on sleep in only the male populations.

3. The authors claim that less sleep in male population is due to aggressive tendencies among male flies. One way to negate or support this hypothesis would be to silence serotonergic PLP neurons that lead to less aggression in flies (Alekseyenko et al., 2014). Such an experiment would involve a simple genetic cross to obtain the correct genotype for further behavioral analyses. Without such a piece of evidence, however, it would be premature to make such claims.

4. In a similar vein, the author's claim that there might be hyperarousal in Amn mutants to explain their behavior results seems speculative and possibly a case of post-hoc reasoning.

Minor concerns/suggestions;

1. More consistent use of asterisks throughout the figures to indicate statistical significance would be helpful to focus on the important results.

2. Constructing a "sleep space" made of 10 dimensions (5 parameters measured in Fig1, each for LP and DP) using Principal component analysis and plotting (i) F individual (ii) M individual, (iii) F population and (iv) M population in this space might be instructive in determining how similar or different each are in their sleep structures.

3. Last line of the abstract (important conclusion line!) is a bit underwhelming. Are the authors interested in making a scientific point rather than a commercial one? Are the authors trying to frame this ms. as a technical study on the benefits of LAM25H? If so, I am a bit confused by the layout of the ms.

4. Normally higher levels of total sleep correspond to lower levels of activity. However, in Fig. 5C&amp;D: both 'total sleep' and 'activity' for striped bars (LP) are higher than the solid bars. This seems strange. No explanation is given.

5. Figure 6 A&amp;B are poorly constructed. With the present color/line-width scheme, it is difficult to interpret. Perhaps a multiple color scheme might work better in this case to increase resolution.

Reviewer 2:

Nearly all fly sleep labs study sleep in single flies under standard conditions (food, 12/12 LD cycle). Nevertheless, there is growing appreciation for the need to understand sleep in different contexts. This manuscript defines a system for examining the influence of social interactions on sleep. While it is primarily a methods paper, the authors also provide a fascinating results indicating that the olfactory system is essential for population effects on sleep.

In my opinion the manuscript can be published as is, but I have provided detailed comments that I hope will be useful to the authors.

The study of sleep in Drosophila is growing rapidly, in part due to well-defined behavioral assays and the availability of commercially available systems for behavioral analysis. There is growing appreciation for complexity of sleep and the limitations of examining sleep in single flies under standard conditions. By examining sleep in populations of flies, the authors present a system to study interactions between sleep and social behavior using commercially available systems. This manuscript is well written and the experiments are technically sound. This study will be of broad interest and the approaches will be utilized by many groups studying social behavior and sleep. All comments below are minor, with the aim of improving the clarity and accessibility of this manuscript.

1. It might be useful to include a diagram of a tube with IR-detector locations for the DAM2 and LAM25H monitors, as many readers will not be completely familiar with these devices. Many more fly labs likely have the single-channel Drosophila Population Monitor. It would be worth mentioning these devices in the discussion as a comparable system to study sleep in populations.

2. Is it fair to say that the method used for calculating sleep (all flies are asleep in populations) dramatically underestimates sleep? I presume that there are ways to model the percentage of flies asleep at any given time, based on the amount of activity, though this certainly has its own limitations. If the authors agree this is a possibility, it would be helpful to add discussion of how modeling could be used to interpret sleep data. An alternative way to look at this might be to take 32 flies in a DAM2 and compare them in total to 32 flies in a single LAM25H tube.

3. In general, more description in the Materials and Methods section would be helpful. Specifically with regards to the mating procedures and sleep calculations. The methods section states sleep parameters were analyzed using MATLAB Software. What is the script? Is it available and has it been used before? If so, a citation would be helpful, or at least stating 'based on parameters defined in xxxx.' Additionally, I understand beam breaks are calculated per population but it would be helpful to clearly spell this out in the methods section.

4. One experiment that may be particularly useful would be to measure single flies in the LAM25H monitors. Many of the findings in Figure 1 are difficult to interpret because it is unclear whether differences observed are due to arena size, beam #/position, or population. This is not a concern for the subsequent figures, and does not impact the overall conclusions of the paper, but a comparison of single flies in DAM2 and LAM25H may help address these differences.

5. In figure 3 the authors find a reduction in sleep followed by robust rebound. As the authors cite, Keene et al, 2010 reported a mild, but significant rebound. It is also important to note that none was observed in Thimgan et al, 2010. It might be fair/useful to highlight the robustness of the rebound in the population-based assay, as it suggests this system may be a more effective means of looking at homeostasis linked to feeding state.

6. On page 9 (1st paragraph) the authors state accurate determination of sleep patters would require tracking software that is not available. This is true, but there are a number of manuscripts describing tracking software that could be used towards this end and are worth describing e.g. Branson et al, 2009; Swierczek et al, 2011; Ardekani et al, 2013

7. In Figure 5 would it be possible to include a calculation of activity/fly to gain insight into how population density is impacting activity? This would seem a more meaningful readout than total activity.

8. The authors fully report all statistics in tables associated with figures. This sets an excellent standard for the field.

Minor comments on figures:

1. In Figure 1, It would be helpful to place legend at the top of the figure. I spent some time looking for it before I noticed it in the bottom right-hand corner. It may even be worth placing on each figure or at least each row.

2. Consider writing 'Dark' or ZT0-12 instead of DP, as this is an abbreviation readers may be less familiar with.

3. In Figure 2, There is a lot of red that makes it difficult to decipher. Slightly modifying the color scheme or thickening the boarders may improve clarity.

4. In Figure 5, line thickness is used to differentiate between group size. This is a very difficult to read and alternative colors would be much more useful.

We appreciate thoughtful reviews and suggestions of the two reviewers as well as the summary of the reviewing editor. Below, we provide our point-by-point response indicating what we have done to address the comments. We believe we have fully addressed all the summary comments. We have also addressed the majority of reviewers comments, allowing us to improve the clarity of this manuscript substantially.

Synthesis of Reviews:

Synthesis Statement for Author (Required):

Both reviewers agree that the manuscript would be an important addition to the Drosophila community studying sleep. They concur that the manuscript should be accepted with minor revisions requiring editor's review. The required revisions are as follows.

1. There should be a better description of the paradigm used (including diagrams of the set up, and an account of software routines used for analysis), with explicit discussion of caveats for each conclusion reached.

We added a new Figure 1 with diagrams of the apparati to explain how the set up detects the activity of flies. Accordingly, Figures 1-8 are now Figures 2-9. We also added the reference for the MatLab analysis program (Page 3, paragraph 2, lines 3-5).

We have tried to make the limitations of the methods and their interpretations more apparent throughout the manuscript (see for example page 5 paragraph 1 line 6-7).

2. Use asterisks consistently throughout the figures to denote significance, following eNeuro rules.

In many of our experiments there are multiple variables and for many of the important conclusions in this paper, comparisons of a data point to multiple other data points is critical. In such analyses, it is impractical and very messy to use asterisks, because there are too many comparisons (see below where we show what would happen to Figure 9G if we used asterisks). The "significance group" annotation is valid, simple and clear. We prefer to retain it except in cases where there is a direct comparison between two groups, as shown in the sleep deprivation experiment (Figure 3) and starvation induced sleep loss and rebound experiment (Figure 4).

3. Discuss the discrepancy with the notion that increased total sleep is accompanied by lower activity in Fig. 5C and D.

The activity measurements we present reflect activity levels ONLY during wake periods, not total activity. It is therefore a metric of locomotor performance, not a measure of how much the fly moved during the light or dark period. Changes in sleep would have no influence on this measure, only changes in intrinsic locomotor processes. A fly who sleeps more could be either hyper (or hypo) locomotor during wake periods. We have tried to clarify this in text (Page 6, paragraph 3, lines 2-5) and changed the labels on the figures from "Activity" to "Activity while awake" in Figures 2D, 5D, 6D and 7D).

4. Use a multiple color scheme to increase the readability of Fig. 6A, B

We changed the line thickness to multiple colors in Figures 7A, B as well as the color scheme for the bar graphs in Figures 7C-F.

5. There are a few unsupported claims (e.g. link between aggression and diminished sleep; hyperarousal in amn mutants), which should be properly placed as speculative statements or eliminated.

We have made it clear in the revised manuscript that these are merely (interesting) speculations. See page 4, paragraph 3, line 13-14, page 6, paragraph 3, lines 12, page 8 paragraph 2, line 5, and page 9, paragraph 4, line 9.

6. There are several other useful comments by reviewers (e.g. construction of a "sleep space" plot for comparisons, rewrite last sentence of abstract, discussion of sleep time calculation, etc.) that would improve the manuscript and these should be considered by authors.

We have addressed the reviewers' specific comments below and we have tried to follow their very useful recommendations as much as possible to improve the manuscript.

Reviewers comments:

Reviewer 1:

In this study, the authors record and analyze sleep behavior in a population of fruit flies. The authors suggest that there are both similarities and differences in sleep characteristics between individual flies and a population of flies. They also suggest that the LAM25H system can be used as a method to investigate neural mechanisms of population behavior.

Admittedly, the described technique to measure sleep in fly populations has its own limitations, especially in the interpretation of sleep structure metrics from populations. If some of the underlying concerns of data interpretation are resolved, this study would be a step forward in understanding the dynamics of sleep in a population of animals. Overall, the study described in this ms. is one that would be useful to the Drosophila sleep community.

Below, I have listed some major and minor concerns with this ms. These include some questions/suggestions that the authors might consider to further improve the ms.

Major concerns;

1. I can't help wonder whether activity in a population of flies truly reflects population behavior or whether it is simply the result of activity of one or few flies that are outliers. The authors admit that their behavioral paradigm cannot distinguish between these possibilities and this is a serious limitation of the paper. However, I am convinced that the presented evidence (especially the sex-specific differences in populations of flies) strongly suggest that population effects on sleep are real. Perhaps, the ms. would greatly benefit with a better description of the paradigm and a better explanation of the caveats in the results. For instance, the authors mention that there are 9 beams across the center of each vial to detect activity, but no explanation is given as to how this achieves a better resolution of fly locomotion and population behavior. A diagram of the paradigm might be a useful addition.

The potential for "rogue" flies to skew the data is a very real concern as we note. During the course of these studies we did some video recordings of populations to try to see if this type of behavior was likely to play a major role in assessment of population sleep. Our conclusion was that it was not- the activity in the populations almost always involved many individuals, not just one active animal. We have mentioned this as "data not shown" on page 9, paragraph 1, lines 8-11.

As for the details of the apparatus, we have included a diagram in the revised manuscript to allow the reader to see how data are collected.

2. In my opinion, the most interesting result in this study has to deal with the population effect on sleep in male flies. Perhaps the ms. would be less complex to follow and more interesting if the subsequent analyses are focused on sleep in only the male populations.

Since this paper is really a first pass at this type of analysis we felt that it was important to explore a variety of factors so we prefer to leave in the female and mixed population data.

3. The authors claim that less sleep in male population is due to aggressive tendencies among male flies. One way to negate or support this hypothesis would be to silence serotonergic PLP neurons that lead to less aggression in flies (Alekseyenko et al., 2014). Such an experiment would involve a simple genetic cross to obtain the correct genotype for further behavioral analyses. Without such a piece of evidence, however, it would be premature to make such claims.

This would be a very interesting experiment, but beyond the scope of this paper. We were actually not trying to claim a role for aggression, but rather suggest it as an interesting possibility. We have reworded the text (Page 4, paragraph 4, line 13-14) to make it clear that this is completely speculative.

4. In a similar vein, the author's claim that there might be hyperarousal in Amn mutants to explain their behavior results seems speculative and possibly a case of post-hoc reasoning.

Again, we have reworded the manuscript (Page 9, paragraph 4, line 9) to make it clear that this is just a speculation.

Minor concerns/suggestions;

1. More consistent use of asterisks throughout the figures to indicate statistical significance would be helpful to focus on the important results.

As noted in the reply to the summary review, for many of the important conclusions in this paper, comparisons of a data point to multiple other data points is critical. In such analyses, it is impractical and very messy to use asterisks, because there are too many comparisons (see below where we show what would happen to Figure 9G if we used asterisks). The "significance group" annotation is valid, simple and clear. We prefer to retain it except in cases where there is a direct comparison between two groups, as shown in sleep deprivation experiment (Figure 3) and starvation induced sleep loss and rebound experiment (Figure 4).

2. Constructing a "sleep space" made of 10 dimensions (5 parameters measured in Fig1, each for LP and DP) using Principal component analysis and plotting (i) F individual (ii) M individual, (iii) F population and (iv) M population in this space might be instructive in determining how similar or different each are in their sleep structures.

That would indeed be interesting but there is a substantial chance that it could be very misleading. For populations and individuals, the parameters we extract do not measure the same things e.g. there are issues with the interpretation of bout length in populations since you are measuring a minimum for the population not the mean of the individual. This is something we will think about for a future paper, but the complexities of interpreting and validating it mean that it would be a substantial amount of work to develop a useful and believable analysis of this type.

3. Last line of the abstract (important conclusion line!) is a bit underwhelming. Are the authors interested in making a scientific point rather than a commercial one? Are the authors trying to frame this ms. as a technical study on the benefits of LAM25H? If so, I am a bit confused by the layout of the ms.

We have revised the abstract to hopefully make this a scientific point (Page 1).

4. Normally higher levels of total sleep correspond to lower levels of activity. However, in Fig. 5C&amp;D: both 'total sleep' and 'activity' for striped bars (LP) are higher than the solid bars. This seems strange. No explanation is given.

The activity measurement reflects activity during wake periods, not total activity. It is therefore a metric of locomotor performance, not a measure of how much the fly moved during the light or dark period. Changes in sleep would have no influence on this measure, only changes in intrinsic locomotor processes. A fly who sleeps more could be either hyper (or hypo) locomotor during wake periods. We have tried to clarify this in text (Page 6, paragraph 3, line 2-5) and changed "Activity" to "Activity while awake" in Figure 2D, 5D, 6D and 7D.

5. Figure 6 A&amp;B are poorly constructed. With the present color/line-width scheme, it is difficult to interpret. Perhaps a multiple color scheme might work better in this case to increase resolution.

We have modified Figure 7 to increase readability.

Reviewer 2:

Nearly all fly sleep labs study sleep in single flies under standard conditions (food, 12/12 LD cycle). Nevertheless, there is growing appreciation for the need to understand sleep in different contexts. This manuscript defines a system for examining the influence of social interactions on sleep. While it is primarily a methods paper, the authors also provide a fascinating results indicating that the olfactory system is essential for population effects on sleep.

In my opinion the manuscript can be published as is, but I have provided detailed comments that I hope will be useful to the authors.

The study of sleep in Drosophila is growing rapidly, in part due to well-defined behavioral assays and the availability of commercially available systems for behavioral analysis. There is growing appreciation for complexity of sleep and the limitations of examining sleep in single flies under standard conditions. By examining sleep in populations of flies, the authors present a system to study interactions between sleep and social behavior using commercially available systems. This manuscript is well written and the experiments are technically sound. This study will be of broad interest and the approaches will be utilized by many groups studying social behavior and sleep. All comments below are minor, with the aim of improving the clarity and accessibility of this manuscript.

1. It might be useful to include a diagram of a tube with IR-detector locations for the DAM2 and LAM25H monitors, as many readers will not be completely familiar with these devices. Many more fly labs likely have the single-channel Drosophila Population Monitor. It would be worth mentioning these devices in the discussion as a comparable system to study sleep in populations.

We have made a figure illustrating the set up and this is now the new Figure 1.

2. Is it fair to say that the method used for calculating sleep (all flies are asleep in populations) dramatically underestimates sleep? I presume that there are ways to model the percentage of flies asleep at any given time, based on the amount of activity, though this certainly has its own limitations. If the authors agree this is a possibility, it would be helpful to add discussion of how modeling could be used to interpret sleep data. An alternative way to look at this might be to take 32 flies in a DAM2 and compare them in total to 32 flies in a single LAM25H tube.

We agree that it is likely that the LAM25H underestimates individual fly sleep and we mention this on page 9. As for trying to model individual behavior from the population data, we feel that it could be very difficult to model the percentage of flies asleep according to the amount of activity since activity of individuals is not measured in the LAM25H. The LAM25H tube is a 25 mm diameter by 95 mm depth vial, and 9 pairs of beams cover a circular section where one/multiple flies can pass. The system records beam breaks which can be generated by one/multiple flies, so it won't be able to predict how many flies are active at a certain time window. It might be possible to model the percentage flies asleep using video recording as well as multiple tracking programs, but that technique would need to be developed.

A second consideration is that the social behavior within the group significantly influences the amount of sleep. It is not a simple linear extrapolation/sum up effect. This aspect of the population data would be lost in comparisons of 32 individual flies to the number of individual flies in LAM25H system.

3. In general, more description in the Materials and Methods section would be helpful.

Specifically with regards to the mating procedures and sleep calculations. The methods section states sleep parameters were analyzed using MATLAB Software. What is the script? Is it available and has it been used before? If so, a citation would be helpful, or at least stating 'based on parameters defined in xxxx.' Additionally, I understand beam breaks are calculated per population but it would be helpful to clearly spell this out in the methods section.

We have added more detail on fly handling and the reference for the software scripts used in the Materials and Methods section (Page 3, paragraph 2, Line 3-5). We have also added a new figure detailing the beam locations in the different apparati.

4. One experiment that may be particularly useful would be to measure single flies in the LAM25H monitors. Many of the findings in Figure 1 are difficult to interpret because it is unclear whether differences observed are due to arena size, beam #/position, or population. This is not a concern for the subsequent figures, and does not impact the overall conclusions of the paper, but a comparison of single flies in DAM2 and LAM25H may help address these differences.

The suggested experiment is interesting, but it would not be surprising to see different sleep profiles with a single fly from these two systems. In addition, the arena size, beam#/position and other environmental differences between DAM2 and LAM25H are not a concern in the current study. The sleep under population condition should be considered as a whole. In fact, we think bigger arena size, more beams are part of the nature of "population". In future experiments, we will try to develop a system that can track a single fly activity within a population.

5. In figure 3 the authors find a reduction in sleep followed by robust rebound. As the authors cite, Keene et al, 2010 reported a mild, but significant rebound. It is also important to note that none was observed in Thimgan et al, 2010. It might be fair/useful to highlight the robustness of the rebound in the population-based assay, as it suggests this system may be a more effective means of looking at homeostasis linked to feeding state.

We added the reference and discussion of this issue (Page 10, paragraph 1, line 7-10).

6. On page 9 (1st paragraph) the authors state accurate determination of sleep patters would require tracking software that is not available. This is true, but there are a number of manuscripts describing tracking software that could be used towards this end and are worth describing e.g. Branson et al, 2009; Swierczek et al, 2011; Ardekani et al, 2013

We have added references and a deeper discussion of this issue (Page 9, paragraph 3, line 5-12).

7. In Figure 5 would it be possible to include a calculation of activity/fly to gain insight into how population density is impacting activity? This would seem a more meaningful readout than total activity.

We could do this, but it is not really going to allow a better comparison to individuals.

8. The authors fully report all statistics in tables associated with figures. This sets an excellent standard for the field.

Thanks!

Minor comments on figures:

1. In Figure 1, It would be helpful to place legend at the top of the figure. I spent some time looking for it before I noticed it in the bottom right-hand corner. It may even be worth placing on each figure or at least each row.

We added legends on top of Figures 2A and B to increase the readability of figures. We added legends on top of Figures 4A and B as well.

2. Consider writing 'Dark' or ZT0-12 instead of DP, as this is an abbreviation readers may be less familiar with.

Changed the figure labels to Light and Dark in all the figures.

3. In Figure 2, There is a lot of red that makes it difficult to decipher. Slightly modifying the color scheme or thickening the boarders may improve clarity.

We increased the thickness of the sleep curves to increase the readability of the figure.

4. In Figure 5, line thickness is used to differentiate between group size. This is a very difficult to read and alternative colors would be much more useful.

We have redone this figure and used color instead of line thickness to differentiate groups.

## References

[B1] Andretic R, Shaw PJ (2005) Essentials of sleep recordings in *Drosophila*: moving beyond sleep time. Methods Enzymol 393:759-772. 10.1016/S0076-6879(05)93040-1 15817323

[B2] Ardekani R, Huang YM, Sancheti P, Stanciauskas R, Tavaré S, Tower J (2012) Using GFP video to track 3D movement and conditional gene expression in free-moving flies. PLoS One 7:e40506. 10.1371/journal.pone.0040506 22829875 PMC3400653

[B3] Branson K, Robie AA, Bender J, Perona P, Dickinson MH (2009) High-throughput ethomics in large groups of *Drosophila*. Nat Methods 6: 451-457. 10.1038/nmeth.1328 19412169 PMC2734963

[B4] Brent MM, Oster II (1974) Nutritional substitution: a new approach to microbial control for *Drosophila* cultures. Dro Inf Ser 155–157.

[B5] Chi M W, Griffith LC, Vecsey CG (2014) Larval population density alters adult sleep in wild-type *Drosophila melanogaster* but not in *amnesiac* mutant flies. Brain Sci 4: 453-470. 10.3390/brainsci4030453 25116571 PMC4194033

[B6] Cirelli C, Bushey D (2008) Sleep and wakefulness in *Drosophila melanogaster*. Ann N Y Acad Sci 1129:323-329. 10.1196/annals.1417.017 18591491 PMC2715168

[B7] Cirelli C, Gutierrez CM, Tononi G (2004) Extensive and divergent effects of sleep and wakefulness on brain gene expression. Neuron 41:35-43. 14715133 10.1016/s0896-6273(03)00814-6

[B8] Donelson NC, Donelson N, Kim EZ, Slawson JB, Vecsey CG, Huber R, Griffith LC (2012) High-resolution positional tracking for long-term analysis of *Drosophila* sleep and locomotion using the “tracker” program. PLoS One 7:e37250. 10.1371/journal.pone.0037250 22615954 PMC3352887

[B9] Dweck HK, Ebrahim SA, Thoma M, Mohamed AA, Keesey IW, Trona F, Lavista-Llanos S, Svatoš A, Sachse S, Knaden M, Hansson BS(2015) Pheromones mediating copulation and attraction in *Drosophila*. Proc Natl Acad Sci U S A 112:E2829-35. 10.1073/pnas.1504527112 25964351 PMC4450379

[B10] Fujii S, Krishnan P, Hardin P, Amrein H (2007) Nocturnal male sex drive in *Drosophila*. Curr Biol 17:244-251. 10.1016/j.cub.2006.11.049 17276917 PMC2239012

[B11] Hanafusa S, Kawaguchi T, Umezaki Y, Tomioka K, Yoshii T (2013) Sexual interactions influence the molecular oscillations in DN1 pacemaker neurons in *Drosophila melanogaster*. PLoS One 8:e84495. 10.1371/journal.pone.0084495 24367668 PMC3867508

[B12] Hay DA (1973) Effects of genetic variation and culture conditions on the social behavior of *Drosophila melanogaster*. Behav Genet 3:107-119. 4199864 10.1007/BF01067651

[B13] Hendricks JC, Finn SM, Panckeri KA, Chavkin J, Williams JA, Sehgal A, Pack AI (2000) Rest in *Drosophila* is a sleep-like state. Neuron 25:129-138. 10707978 10.1016/s0896-6273(00)80877-6

[B14] Huber R, Hill SL, Holladay C, Biesiadecki M, Tononi G, Cirelli C (2004) Sleep homeostasis in *Drosophila melanogaster*. Sleep 27:628-639. 15282997 10.1093/sleep/27.4.628

[B15] Isaac RE, Li C, Leedale AE, Shirras AD (2010) *Drosophila* male sex peptide inhibits siesta sleep and promotes locomotor activity in the post-mated female. Proc Biol Sci 277:65-70. 10.1098/rspb.2009.1236 19793753 PMC2842620

[B16] Keene AC, Duboué ER, McDonald DM, Dus M, Suh GS, Waddell S, Blau J (2010) Clock and cycle limit starvation-induced sleep loss in *Drosophila*. Curr Biol 20:1209-1215. 10.1016/j.cub.2010.05.029 20541409 PMC2929698

[B17] Krupp JJ, Kent C, Billeter JC, Azanchi R, So AK, Schonfeld JA, Smith BP, Lucas C, Levine JD (2008) Social experience modifies pheromone expression and mating behavior in male *Drosophila* melanogaster. Curr Biol 18:1373-1383. 10.1016/j.cub.2008.07.089 18789691

[B18] Larsson MC, Domingos AI, Jones WD, Chiappe ME, Amrein H, Vosshall LB (2004) *Or83b* encodes a broadly expressed odorant receptor essential for *Drosophila* olfaction. Neuron 43:703-714. 10.1016/j.neuron.2004.08.019 15339651

[B19] Levine JD, Funes P, Dowse HB, Hall JC (2002) Resetting the circadian clock by social experience in *Drosophila melanogaster*. Science 298:2010-2012. 10.1126/science.1076008 12471264

[B20] Linford NJ, Chan TP, Pletcher SD (2012) Re-patterning sleep architecture in *Drosophila* through gustatory perception and nutritional quality. PLoS Genet 8:e1002668. 10.1371/journal.pgen.1002668 22570630 PMC3342939

[B21] Liu W, Guo F, Lu B, Guo A (2008) *Amnesiac* regulates sleep onset and maintenance in *Drosophila melanogaster*. Biochem Biophys Res Commun 372:798-803. 10.1016/j.bbrc.2008.05.119 18514063

[B22] Liu W, Liang X, Gong J, Yang Z, Zhang YH, Zhang JX, Rao Y (2011) Social regulation of aggression by pheromonal activation of Or65a olfactory neurons in *Drosophila*. Nat Neurosci 14:896-902. 10.1038/nn.2836 21685916

[B23] Lone SR, Sharma VK (2011) Social synchronization of circadian locomotor activity rhythm in the fruit fly *Drosophila melanogaster*. J Exp Biol 214:3742-3750. 10.1242/jeb.057554 22031738

[B24] Lone SR, Sharma VK (2012) Or47b receptor neurons mediate sociosexual interactions in the fruit fly *Drosophila melanogaster*. J Biol Rhythms 27:107-116. 10.1177/0748730411434384 22476771

[B25] Mrosovsky N (1988) Phase response curves for social entrainment. J Comp Physiol A 162:35-46. 3351785 10.1007/BF01342701

[B26] Sakai T, Ishida N (2001) Circadian rhythms of female mating activity governed by clock genes in *Drosophila*. Proc Natl Acad Sci U S A 98:9221-9225. 10.1073/pnas.151443298 11470898 PMC55401

[B27] Schneider J, Dickinson MH, Levine JD (2012) Social structures depend on innate determinants and chemosensory processing in *Drosophila*. Proc Natl Acad Sci U S A 109:17174-17179. 10.1073/pnas.1121252109 22802679 PMC3477376

[B28] Shaw J, Cirelli C, Greenspan RJ, Tononi G (2000) Correlates of sleep and waking in *Drosophila melanogaster*. Science 287:1834-1837. 10710313 10.1126/science.287.5459.1834

[B29] Stern K, McClintock MK (1998) Regulation of ovulation by human pheromones. Nature 392:177-179. 10.1038/32408 9515961

[B30] Swierczek NA, Giles AC, Rankin CH, Kerr RA (2011) High-throughput behavioral analysis in *C*. *elegans.* Nat Methods 8:592-598. 10.1038/nmeth.1625 21642964 PMC3128206

[B31] Tauber E, Roe H, Costa R, Hennessy JM, Kyriacou CP (2003) Temporal mating isolation driven by a behavioral gene in *Drosophila*. Curr Biol 13:140-145. 12546788 10.1016/s0960-9822(03)00004-6

[B32] Thimgan MS, Suzuki Y, Seugnet L, Gottschalk L, Shaw PJ (2010) The perilipin homologue, lipid storage droplet 2, regulates sleep homeostasis and prevents learning impairments following sleep loss. PLoS Biol 8:e1000466 10.1371/journal.pbio.1000466 20824166 PMC2930866

[B33] Toma DP, Bloch G, Moore D, Robinson GE (2000) Changes in period mRNA levels in the brain and division of labor in honey bee colonies. Proc Natl Acad Sci U S A 97:6914-6919. 10841583 10.1073/pnas.97.12.6914 PMC18775

[B34] Wang L, Anderson DJ (2010) Identification of an aggression-promoting pheromone and its receptor neurons in *Drosophila*. Nature 463:227-231. 10.1038/nature08678 19966787 PMC2999963

[B35] Wolf FW, Rodan AR, Tsai LT, Heberlein U (2002) High-resolution analysis of ethanol-induced locomotor stimulation in *Drosophila*. J Neurosci 22:11035-44. 12486199 10.1523/JNEUROSCI.22-24-11035.2002 PMC6758417

[B36] Wu MN, Koh K, Yue Z, Joiner WJ, Sehgal A (2008) A genetic screen for sleep and circadian mutants reveals mechanisms underlying regulation of sleep in *Drosophila*. Sleep 31:465-472. 18457233 10.1093/sleep/31.4.465 PMC2279757

[B37] Wu Z, Gong Z, Feng C, Guo A (2000) An emergent mechanism of selective visual attention in *Drosophila*. Biol Cybern 82:61-68. 10650908 10.1007/s004220050006

[B38] Zimmerman JE, Chan MT, Jackson N, Maislin G, Pack AI (2012) Genetic background has a major impact on differences in sleep resulting from environmental influences in *Drosophila*. Sleep 35:545-557. 10.5665/sleep.1744 22467993 PMC3296797

